# Does depression in Alzheimer's disease show evidence of differential gene expression in neurons or microglia compared to Alzheimer's disease without depression?

**DOI:** 10.1177/25424823261472076

**Published:** 2026-08-01

**Authors:** Lindsey I Sinclair, Mizuki Morisaki, Peter P Henley, Emma L Dempster, Clive G Ballard, Jordan T Lin, Robert MacLachlan, Seth Love

**Affiliations:** 1 Cerebrovascular and Dementia Research Group, Bristol Medical School, 7423 University of Bristol, Southmead Hospital, Bristol, UK; 2 Department of Psychiatry, Faculty of Medicine, University of Southampton, College Keep, Southampton, UK; 3 Dementia Research Institute, 2112 University of Cardiff, Cariff, UK; 4 3286 NIHR Exeter Biomedical Research Centre, University of Exeter Medical School, Exeter, UK

**Keywords:** Alzheimer's disease, depression, depressive disorder, gene expression

## Abstract

**Background:**

Depression in Alzheimer's disease (AD) is common, distressing, difficult to treat and inadequately understood.

**Objective:**

We compared gene expression in neurons and microglia from AD patients +/- depression, stratified according to depression polygenic risk score (PRS-D) and looked for differential pathway regulation.

**Methods:**

We identified AD patients with (*n* = 95) or without depression (*n* = 25) whose brains had been donated to a UK brain bank. Exclusion criteria included other disorders known to affect cognition, previous major psychiatric disorder and a neuropathological diagnosis of a non-AD dementia. Depression was defined as a clinical depression diagnosis or Geriatric Depression Scale/Center for Epidemiologic Studies Depression Scale >7. PRS-D was calculated using LDpred2. In the AD + depression group, only the top (*n* = 29) and bottom tertile (*n* = 30) of PRS-D were analyzed by microarray. Tissue from superior frontal gyrus (SFG) and anterior insula (AIns) was sorted into neuronal and microglial fractions, and gene expression measured using a ClariomS Pico microarray.

**Results:**

After quality control, microarray data were available for AIns microglia (*n* = 51), AIns neurons (*n* = 59), SFG microglia (*n* = 60) and SFG neurons (*n* = 63). No individual genes had a *p* < 0.05, adjusted for false discovery rate. Gene set enrichment analysis identified significantly upregulated and downregulated pathways in both brain regions and cell types. More pathways were differentially expressed in microglia than in neurons. Many of these pathways related to infection or inflammation but also included olfactory transduction and Parkinson's disease.

**Conclusions:**

In conclusion we have identified pathways that may contribute to the development of depression in AD. A majority were identified predominantly in microglia.

## Introduction

Memory loss is the best-known symptom of Alzheimer's disease (AD) but behavioral and psychological symptoms affect >50% of people with dementia.^1^ One of the most common of these symptoms is depression, which affects up to 1 in 7 people with AD.^2^ Currently available antidepressants are not effective against depression in AD^3–5^ and other treatment options are very limited. There is an urgent need for new treatments to be developed to treat depression in people living with dementia.

The underlying biology of depression in AD is not known. Some, but not all research, has suggested that it may have a vascular component (e.g.,^2,6,7^). Our recent work and that of others has demonstrated that depression in AD appears to be a different disease process from depression in those without dementia.^8^ This could help to explain why currently available antidepressant treatments are ineffective in treating depression in people living with dementia. RNAseq bulk gene expression data from the ROSMAP study has not shown differences in bulk gene expression between depressed/not depressed people with AD (in submission). We found that depression in AD appears to have different risk factors^9^ from depression itself and that it appears to be associated with differential regional atrophy in frontal brain areas.^10^

Depression in AD was reported to be associated with greater AD severity,^11^ faster progression of the disease^12,13^ and earlier residential placement.^14,15^ There is, however, no consistent evidence that depression in AD or mild cognitive impairment (MCI) is associated with AD-related changes in cerebrospinal fluid amyloid-β and phosphorylated tau.^16^ Some^17,18^ but not all previous studies^19^ found increases in global measures of AD pathology in those with depression in AD. One more detailed study linked depression in AD to greater neuritic plaque density in the temporal lobe.^20^ One (unconfirmed) suggestion is that depression in AD may be due to AD pathology in subcortical nuclei such as the raphé and locus coeruleus.^21^ A meta-analysis including 3 studies (*n* = 352) found a small benefit from selective serotonin reuptake inhibitor treatment on cognitive decline in people with AD.^22^

In recent years genome-wide association studies in depression have allowed the development of polygenic risk scores for depression (PRS-D). Very recently it was shown that a high PRS-D increases the risk of conversion from MCI to AD.^23^ Other authors suggested that depression occurring during MCI may reflect a more aggressive pathology.^24^ Yet genetic risk for AD was not predictive of later-life depression in individuals without AD.^25^

Studying gene expression allows measurement of the activity of thousands of genes, providing much more information than any currently available protein-based assay. This affords a holistic view of what is going on in brain cells at a given point in time and can help to identify proteins that are increased or decreased to a greater extent than others, and which thus might be related to pathology.

We hypothesized that there are differences in gene expression in neurons and microglia from the superior frontal gyrus (SFG) and anterior insula (AIns), between individuals with, and those without depression in AD, and that these differences are greatest in those at lowest genetic risk of depression. We thought it likely that these differences would be most pronounced in neurons. We chose these brain areas based on previous work^10^ which identified that these areas had differential atrophy in individuals with depression in AD.

## Methods

Through interrogation of the UK Brain Banks Network database, we identified 120 brain donations from individuals with a pathological diagnosis of AD who had developed depression during their illness. Tissue used in this study was provided by the South West Dementia Brain Bank, the Newcastle Brain Tissue Resource, the Oxford brain bank and the London Neurodegenerative Diseases Brain Bank. Many of these 120 individuals had been part of the Brains for Dementia Research cohort, but not all. They had either a clear clinical diagnosis of depression during their illness, and/or a Geriatric Depression Scale (GDS) score of ≥8, a Cornell score ≥8, or Neuropsychiatric Inventory-depression ≥2 and frequency ≥2. We chose these thresholds based on our previous work and because these thresholds give the best balance between sensitivity and specificity.^9,26,27^ Individuals with significant depressive illness more than 2 years prior to diagnosis of dementia were excluded, as were individuals with a history of a clinically significant head injury, severe small vessel disease, moderate small vessel disease and previous stroke, a limbic-predominant age-related TDP-43 encephalopathy stage ≥3, neocortical evidence of Lewy body disease or a neuropathological diagnosis of a cause of dementia other than AD. For the control group the same neuropathological and clinical exclusion criteria were applied except that individuals had to have never scored above threshold on the GDS, Neuropsychiatric Inventory-depression subscale, or Cornell, and clinical notes were reviewed to ensure that there had not been a depressive illness during or prior to the dementia. After selection of this group of 120 individuals it unfortunately became apparent that fresh frozen tissue was not available for 12 individuals for the brain areas being investigated in this study. This exclusion of the 12 individuals took place after the PRS had been calculated. This combined with genetic data not being available in a useable format for 9 individuals and additional clinical and neuropathological data being made available after initial cohort selection resulted in a final useable cohort of 95 individuals for whom PRS-D were available (see Figure 1).

**Figure 1.**
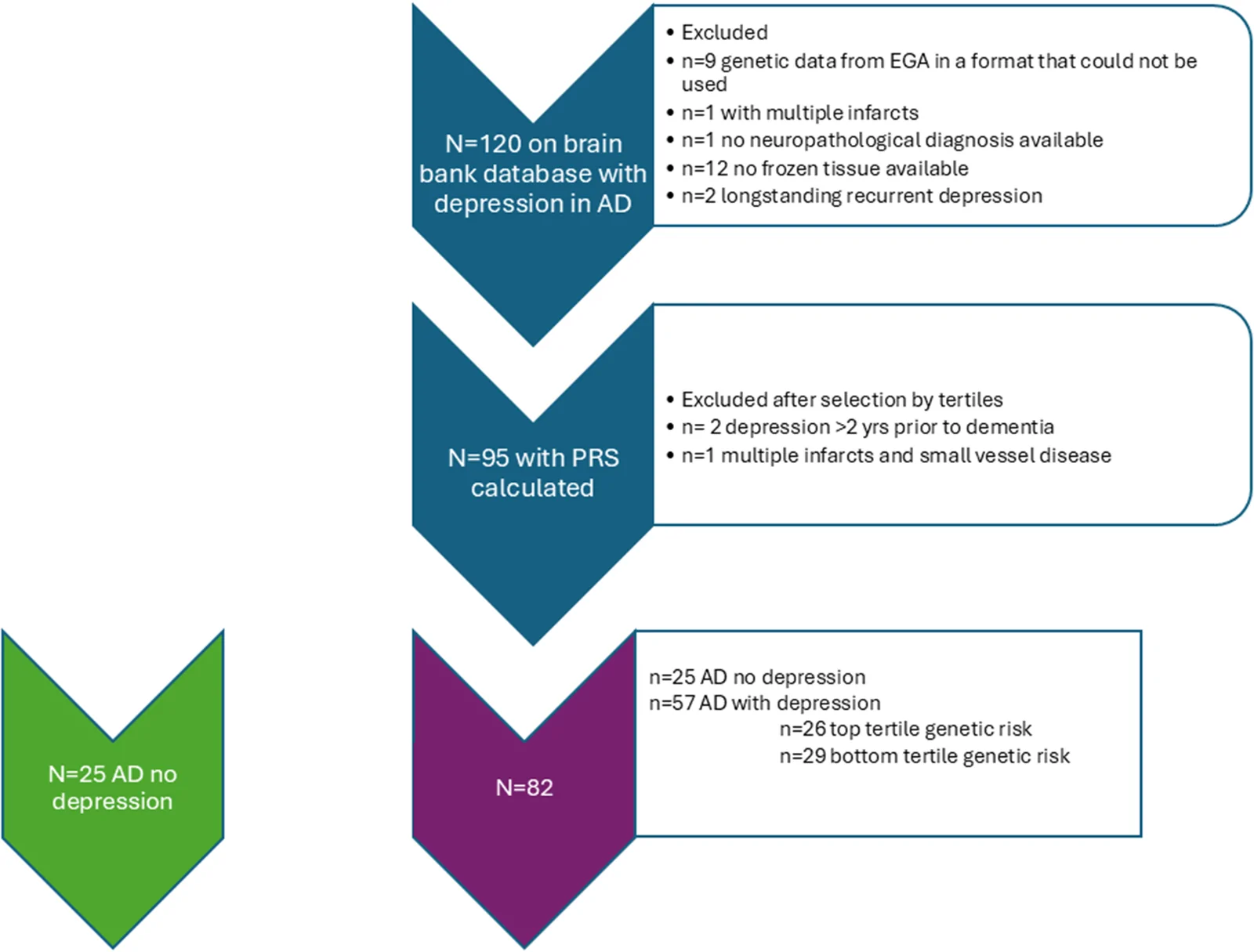
Flowchart for case selection for this study.

### Genotyping and PRS calculation

Where genetic data were not already available, genomic DNA was extracted from 50–100 mg of fresh frozen tissue by using a Kingfisher DNA kit. Genomic DNA was extracted from fresh frozen brain tissue from the SFG for each sample. Sample concentrations were measured using a Qubit DNA kit (ThermoFisher Scientific). After quality control (QC) by nanodrop and sample dilution, individuals were genotyped using a UKBiobank axiom array in the University of Bristol genomics facility. Unimputed genetic data were cleaned in PLINK 1.9 (using thresholds of individual missingness 0.02, genotype missingness of 0.02, minor allele frequency of 0.05, Hardy-Weinberg Equilibrium 10^6^, relatedness of 0.2). We used the MDS method for population stratification, using the 1000 genomes reference panel^28^ and all individuals were of European ancestry. The data was then imputed using the Michigan Imputation server v1 with 1000 Genomes Phase 3 v5 as the reference panel.^29^ We used the same imputation method as the Brains for Dementia Research (BDR) imputed genetic data which were already available to us through the UK brain bank network.^30^ After QC of the newly imputed data in PLINK 2.0 using the same threshold as above and excluding SNPs with an imputation information score of <0.5, the data were combined in PLINK 1.9 with the BDR imputed genetic data, as this contained the genotype information for the samples that had already been genotyped. This combined dataset was then re-cleaned, re-applying the previous thresholds for missingness, minor allele frequency, and Hardy-Weinberg Equilibrium. The PRS-D was calculated in R using LDpred2 (bigsnpr)^31^ with the Hapmap3+ SNPs described by Privé et al. Summary statistics from the Howard et al. GWAS were used^32^ in generating this PRS score and the NACC cohort was used as a reference genetic population. The same QC steps with the same thresholds were applied to the NACC genetic dataset. We confirmed using both demographic and genetic data (multidimensional scaling (MDS) approach using 1000 genomes as a reference population^28^) that the ancestry of the NACC cohort matched the cohort used in this study. Only individuals with European ancestry in the MDS analysis from the NACC cohort were included in the genetic dataset used as a reference population, as this best matched the cohort used in this study. After PRS calculation, the PRS scores for the samples in the depression in AD cohort for this study were extracted and divided into tertiles. The top and bottom tertile of polygenic risk for depression (in the depression in AD group) were selected for further study. Tissue was obtained from the SFG (medial segment) and the AIns for these individuals. Where tissue was not available, the individual with the next nearest PRS-D in the middle tertile was chosen as a replacement. There were 6 such replacements made. Tissue was obtained from both brain areas for the “control” group (*n* = 25) of donors who had had AD but not depression.

### Tissue homogenization

Approximately 300 mg of fresh frozen tissue per individual per brain area was homogenized on ice in 2 ml of EZ lysis buffer (Sigma Aldrich) using a Dounce homogenizer. After 5 strokes with the loose pestle and 5–10 strokes with the tight pestle the sample was inspected to ensure that homogenization was complete. The sample was then vortexed briefly before a further 2 ml of EZ lysis buffer was added. After 5 min of incubation on ice the sample was spun for 5 min at 500 g at 4°C. The supernatant was removed, the pellet re-suspended in 1 ml of EZ lysis buffer and a further 3 ml of EZ lysis buffer added. The sample was then spun for 5 min at 500 g at 4°C. The supernatant removed and the pellet suspended in 1 ml of sample buffer (0.5% BSA/PBS). 500 µl of 2.2 M sucrose was added to a sterile 2 ml Eppendorf tube, then 500 µl of sample and 900 µl of 2.2 M sucrose. The sample was spun for 45 min at 14,000 g at 4°C. After centrifugation, the supernatant and any myelin debris were removed. The pellet was re-suspended in 500 µl of sample buffer and a further 500 µl of sample buffer was added. After a final spin for 5 min at 500 g at 4°C the supernatant was again removed and the pellet re-suspended in 500 µl of stem cell banker (Amsbio) and frozen at −80°C until required.

### Fluorescence-activated nuclei sorting (FANS)

For immunolabelling, the samples were defrosted and then re-suspended in staining buffer (0.5%BSA/PBS with 5 µl/ml RNAse inhibitor) before being spun at 1500 g at 4°C for 5 min. The supernatant was discarded and the pellet re-suspended in 1000–500 µL of staining buffer. Hoechst dye was added at 1 in 500 and 50–100 µl of the sample was removed to a separate sterile Eppendorf tube which already contained remaining staining buffer as the unlabeled control. A further 50–100 µl was added to the remainder of the sample, to which NeuN antibody 1:500 (Millipore MAB377X) and IFR8 antibody 1:100 (Invitrogen 17-9852-82) were then added. Both the labelled and unlabeled samples were incubated for 90 min at 4°C on a rotor in the dark. The labelled sample was spun at 4°C at 1500 g for 5 min and the supernatant was removed. The pellet was re-suspended in 1 ml of fresh staining buffer and kept on ice during transport to the flow cytometry facility. Immunolabelling and cell separation were performed in batches of 3 samples in the University of Bristol flow cytometry facility by use of a BD aria flow cytometer (BD Biosciences) with a 70 µM nozzle and a threshold of forward scatter value of 500. The thresholds were set according to the work of our collaborator Emma Dempster.^33,34^ Separated cell fractions were collected directly into trizol in a sterile tube and frozen at −80°C until required.

### RNA extraction

RNA extraction on cell fractions was performed with a microzol direct kit (Zymo), at room temperature, as per the manufacturer's instructions. Samples were eluted in 10 µl of sterile water and then frozen at −80°C until required. Care was taken to keep the number of freeze/thaw cycles <3. RNA concentration was measured using a Qubit high sensitivity kit (ThermoFisher Scientific) and RNA integrity number (RIN) was measured using an Agilent bioanalyzer with a pico bioanalyzer kit (Agilent).

### Microarray

For the NeuN samples, all RNA was diluted to 3.3 ng/µl and the IFR8 samples were diluted to 2 ng/µl. RNA was transported on dry ice to the University of Bristol genomics facility. Gene expression was measured using a Clariom S pico microarray kit (Thermo Scientific), as per the manufacturer's instructions. For the SFG NeuN and IRF8 samples 7 amplification cycles were performed, but for the insula 6 amplification samples were performed. Sample QC was performed by nanodrop during microarray sample preparation. We chose to assess gene expression using microarray as the RNA in this study did not meet the threshold required for RNAseq to be used, primarily due to the RIN values obtained. All samples for each cell type in each brain area were assayed on the same microarray plate; therefore, we did not include batch effects in our analyses.

### Statistical analysis and data cleaning

Data cleaning and statistical analyses were performed in either STATA or R [version 4.4.0]. The PRS were generated using the bigsnpr R package.^31^ For statistical tests *p* < 0.05 was deemed significant, with appropriate adjustments made for multiple testing in the gene expression analysis.

### Differential expression analysis

In our power analysis prior to the study, we had calculated that a standard deviation of 0.80 and a sample size of 30 per group (1/4 of 120) would give 90% power to detect a 2-fold difference in gene expression.^35^ The primary outcome was gene expression in the NeuN cell fraction from AD brain donors with depression, compared to AD donors without depression. Secondary outcomes included gene expression in the IRF8 cell fraction and differences between the top and bottom tertile of PRS-D compared to controls.

The differential expression analysis and pre analysis QC checks were performed in the ‘Transcriptome Analysis Console’ (TAC) [ThermoFisher Scientific, version 4.0], which measures the expression of 21,448 genes, including protein coding and non-coding transcripts. Our QC process excluded samples that failed the AUC threshold and were clear outliers on PCA. For further details and justifications for the samples excluded please refer to the Supplemental Material. For the differential expression analysis the TAC default algorithm settings were used, with the ‘gene level SST-RMA’ summarization method selected. Covariates included age at death, sex, Braak tangle stage, postmortem delay, and RIN score. To adjust for multiple testing, genes with an outputted FDR *p* value <0.05 were deemed differentially expressed. For details on the sample QC, see the Supplemental Methods.

To assess the aggregative effect of multiple gene expression changes on biological processes we performed ‘gene set enrichment analysis’ (GSEA) using ClusterProfiler^36^ [R Package, version 4.12.5], based on the known gene assignments for 317 biological pathways allocated in the KEGG database (https://www.genome.jp/kegg/pathway.html). As opposed to standard enrichment approaches which only take genes reaching pre-determined thresholds for statistical significance and sometimes fold changes, GSEA significance testing is based on all the genes included in the differential expression analysis being either upregulated or downregulated in a given pathway. Specifically, this is tested by first ranking the gene expression associations with the condition being tested from the most positively associated to the most negatively associated, and then scoring each gene within these biological pathways by the extent that it is upregulated or downregulated in this ranked list. The statistical significance of the sum of the ranked gene positions for each pathways being upregulated or downregulated is then tested against permuted data.^37^

As we had no individual genes that met the threshold for statistical significance standard enrichment analysis would not have been appropriate for this dataset. Our GSEA workflow removed genes that were not flagged as expressed in either of the conditions being tested in each analysis, as well as genes that did not have an Entrez ID annotation. Since genes with low expression have higher relative variance, we then removed genes that were within the lowest 20^th^ quantile for expression levels in both the conditions being tested, to avoid spurious fold changes influencing the analysis. For the GSEA settings, the minGSSize setting (minimum number of genes in each tested pathway) was set to 20, and pathways with an outputted Benjamini Hochberg-adjusted *p* values <0.05 were deemed as significantly enriched. For the secondary analysis an additional Bonferroni adjustment was made to these *p* values to account for the three PRS comparisons.

For GSEA input, we used the linear space fold changes (which control for covariates) generated from the differential expression analysis, after the filtering steps described above were applied. Since some fold changes were tied, the fold changes were secondly ranked with lower *p* values ranked higher for upregulated genes and lower *p* values ranked lower for more downregulated genes, since the most upregulated and downregulated genes are at the top and bottom of the list, respectively.

To additionally test that our findings were robust when alternative ranking metrics were used, for all the GSEA carried out, we additionally performed a confirmatory GSEA which was based on statistical significance. Here the data was reformatted, such that the most significantly upregulated genes were at the top and the most significantly downregulated genes at the bottom of the ranked list. Conversely, here *p* value ties were then broken based on fold changes. In terms of the complete analysis, the first set of GSEAs (based on fold changes) were presented as our findings; however, any pathways that could not be confirmed in the second analysis were removed, thus, our findings are based on a higher weighting for effect size and verified based on significance.

## Results

After applying our exclusion criteria and after fill-in genotyping as described above and combination of these data with the existing BDR genotype data, there was sufficient genetic data for analysis of 95 AD donors who met the depression caseness criteria. Forty-seven of these individuals were female and 48 were male. The distribution of the PRS scores is shown in Supplemental Figure 1. Division into tertiles of genetic risk placed 30 individuals in the bottom tertile and 29 in the top tertile of genetic risk. We then made a further tissue request so that we had 0.5 g of fresh frozen tissue from the SFG and AIns from all individuals in the top and bottom tertile of genetic risk of depression. At this stage (see Figure 1), 3 individuals had to be excluded and 3 had no frozen tissue available.

Ultimately, after exclusions due to lack of tissue availability, tissue from 82 AD patients was used for the microarray component of this study: 25 who had not been depressed during their illness and 57 who had developed depression. Two of the AD + depression group, who were included as they had been used in a previous project using this cohort,^38^ did not have PRS information available. There was no evidence of a between-group difference in age at death, Braak tangle stage, Braak Lewy body stage, sex or medication use in the last 3 months prior to death. There was however a between-group difference in postmortem delay, as shown in Table 1.

**Table 1.** Clinical, pathological. and demographic information for the individuals included in this study.

	AD no depression *n* = 25	AD depression total *n* = 57	Statistical evidence (Mann-Whitney test, *χ*^2^ test or *t*-test)	Bottom tertile of genetic risk *n* = 29	Top tertile of genetic risk *n* = 26	Statistical evidence (Mann-Whitney test, *χ*^2^ test or *t*-test)
Age at death (y)	82.8 (7.8)	82.7 (8.6)	*p* = 0.948	82.3 (8.5)	83.4 (9.3)	*p* = 0.634
Postmortem interval (h)	30.2 (14.6)	44.3 (25.5)	*p* = 0.016	41.4 (29.5)	46.1 (21.2)	*p* = 0.283
Sex			*χ*^2^ = 2.70, *p* = 0.100			*χ*^2^ = 0.356, *p* = 0.551
* Female*	10	34		19	15	
* Male*	15	23		10	11	
Duration of AD (y)	7.3 (4.2)	8.6 (3.0)	*p* = 0.203	9.4 (3.1)	8.3 (2.7)	*p* = 0.320
Braak stage			*χ*^2^ = 0.274, *p* = 0.965			*χ*^2^ = 1.032, *p* = 0.794
* *III	2	3		1	2	
* *IV	1	2		1	1	
* *V	8	20		9	10	
* *VI	14	32		18	13	
Braak Lewy body stage			*χ*^2^ = 3.667, *p* = 0.300			*χ*^2^ = 3.340, *p* = 0.342
* 0*	17	36		17	18	
* Amygdala only*	3	2		2	0	
* 2*	0	1		0	1	
* Unclassifiable*	0	3		1	2	
*APOE* genotype			*χ*^2^ = 8.332, *p* = 0.080			*χ*^2^ = 1.061, *p* = 0.787
* *3-Feb	2	0		0	0	
* *4-Feb	3	3		2	1	
* *3-Mar	7	14		9	5	
* *4-Mar	5	14		6	7	
* *4-Apr	0	6		3	2	
Last MMSE	14.8 (8.0)	11.2 (8.6)	*p* = 0.142	11.5 (9.5)	10.5 (7.9)	*p* = 0.703
Antidepressant use in last 3/12			*χ*^2^ = 17.48, *p* < 0.001			*χ*^2^ = 0.138, *p* = 0.711
* No*	20	16		8	8	
* Yes*	5	38		20	16	
Antipsychotic use in last 3/12			*χ*^2^ = 0.614, *p* = 0.433			*χ*^2^ = 0.021, *p* = 0.884
*No*	20	38		20	16	
*Yes*	5	15		8	7	
Anti-inflammatory use in last 3/12			*χ*^2^ = 0.675, *p* = 0.411			*χ*^2^ = 0.144, *p* = 0.704
* No*	7	21		12	9	
* Yes*	16	31		15	14	

Microarray and GS*E*A results

For each individual analysis, microarray gene expression quantification on samples from the following number of individuals: 60 (AIns Microglia), 65 (AIns Neurons), 62 (SFG Microglia), and 65 (SFG Neurons). All samples for each brain area and cell type were run on the same plate, to reduce variability. Sample level QC excluded a further 9 samples from the AIns microglia analysis, 6 from AIns neurons, 1 from the SFG microglia, and 2 from the SFG neurons (for details of the sample level QC exclusions and justifications, see the Supplemental Methods). Ultimately, for each analysis, the AIns microglia comprised 17 controls and 34 depression cases, of which 17 individuals belonged to each of the upper and lower PRS tertiles. The AIns neurons comprised 21 controls and 38 depression cases, of which there were 19 individuals for each PRS tertile. The SFG microglia comprised 18 controls and 42 depression cases, of which 21 cases were in the top tertile and 21 in the bottom. The SFG neurons comprised 20 controls and 43 depression cases, with 22 and 21 cases in the top and bottom tertiles respectively.

Out of the 21,448 gene expression changes quantified on the microarrays, no individual genes were significantly differentially expressed in neurons and microglia in any of the brain regions in patients with AD + depression, nor were there any significant expression differences across all the PRS conditions tested in our secondary analysis. This may reflect sample size, or heterogeneity in the underlying neurobiology of depression in AD, but could also reflect a genuine lack of any hallmark gene expression differences with the AD + depression phenotype. Nonetheless GSEA identified multiple significantly upregulated and downregulated pathways in both brain regions and cell types, as well as in most of the PRS conditions in the secondary analysis, which strongly indicates that some subtle changes to gene expression patterns are more common in AD + depression, with some of these changes potentially being attributed to genetics. When performing GSEA, genes were removed that could not be annotated to Entrez IDs, or that had low or absent expression (see methods for details). As such, each GSEA was based on a subset of the overall genes quantified on the TAC console (see Supplemental Material 2 for further details).

### Anterior insular cortex microglia

In the AIns microglia, 3 pathways were significantly upregulated and 2 downregulated in the depression cases (See Figure 2 and P1: Supplemental Material 2). In the top depression PRS group versus control 3 pathways upregulated and 2 pathways were downregulated. In the bottom depression PRS versus control, two pathways were upregulated and five downregulated.

**Figure 2.**
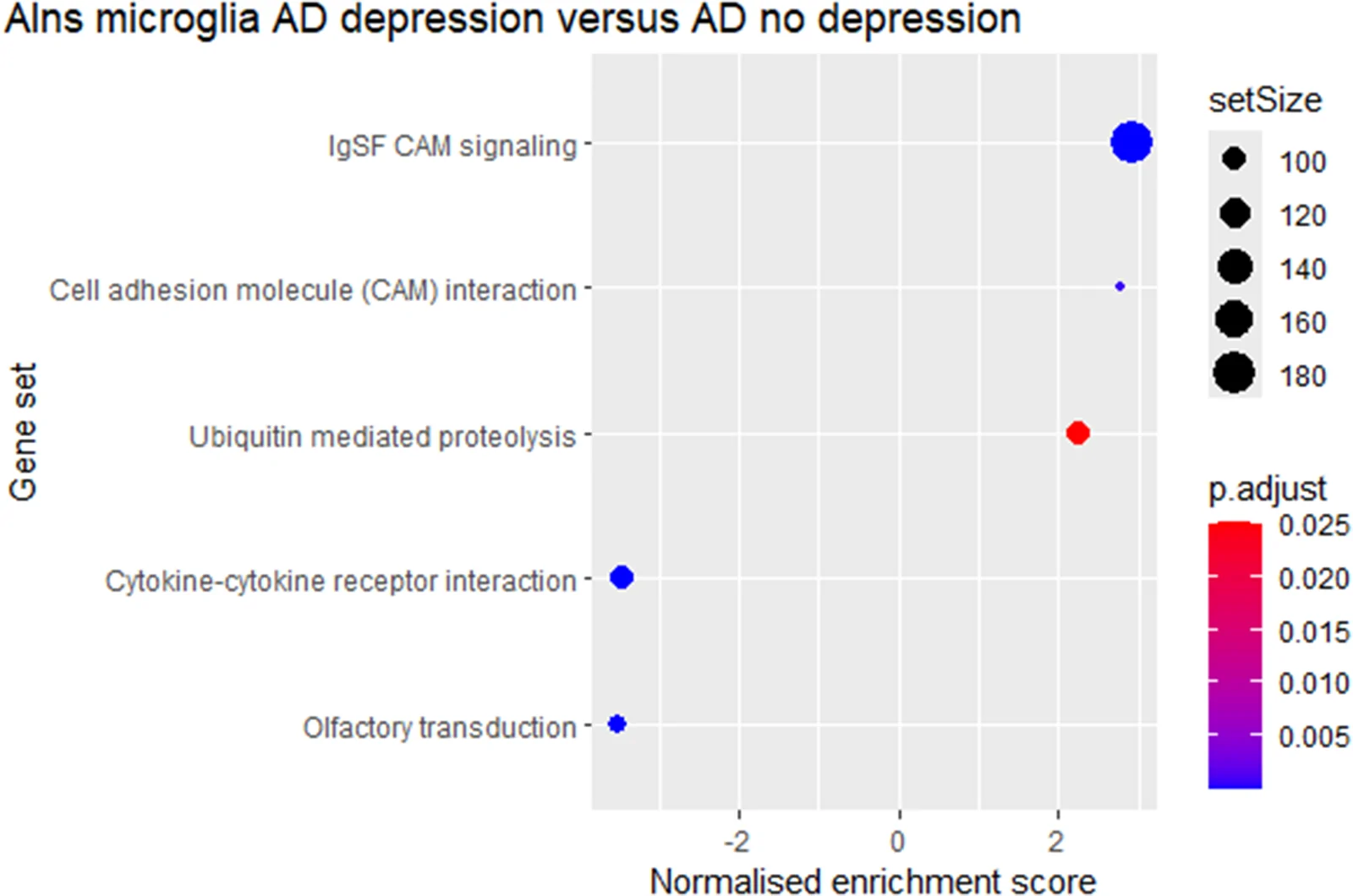
Visualization of the gene set enrichment analysis for the AIns microglial gene set, comparing all of the individuals with AD and depression to the AD without depression group.

Interestingly in the top PRS analysis versus control, the additional pathway that was not upregulated in the bottom PRS versus control was Parkinson's disease. When comparing the PRS groups to each other 3 pathways were upregulated in the top PRS tertile, which again indicated that expression changes related to Parkinsons disease pathways might be influenced by PRS risk for depression, as well as prion disease and neuroactive ligand-receptor interaction. In the bottom PRS group, two pathways were upregulated, including spliceosome and ATP dependent chromatin remodeling, PRS results are shown in Supplemental Figure 7 and P1: Supplemental Material 2.

### Superior frontal gyrus microglia

In SFG microglia, 7 pathways were upregulated and 62 downregulated in the depression cases (See Figure 3 and P3: Supplemental Material 2). Many of the downregulated pathways were related to responses to infections, e.g., Shigella and Salmonella. In the PRS analysis more pathways were upregulated and downregulated in the top PRS group versus AD no depression than in the bottom PRS group versus AD no depression: 6 pathways upregulated and 58 pathways downregulated in the top PRS tertile, and 2 upregulated and 15 downregulated in the bottom PRS tertile. Interestingly olfactory transduction was upregulated in both PRS groups, and others, e.g., Salmonella infection, were downregulated in both PRS groups. When comparing the PRS groups to each other, only olfactory transduction was upregulated in the top PRS tertile and no pathways were downregulated. PRS results are shown in Supplemental Figure 8 and P3: Supplemental Material 2.

**Figure 3.**
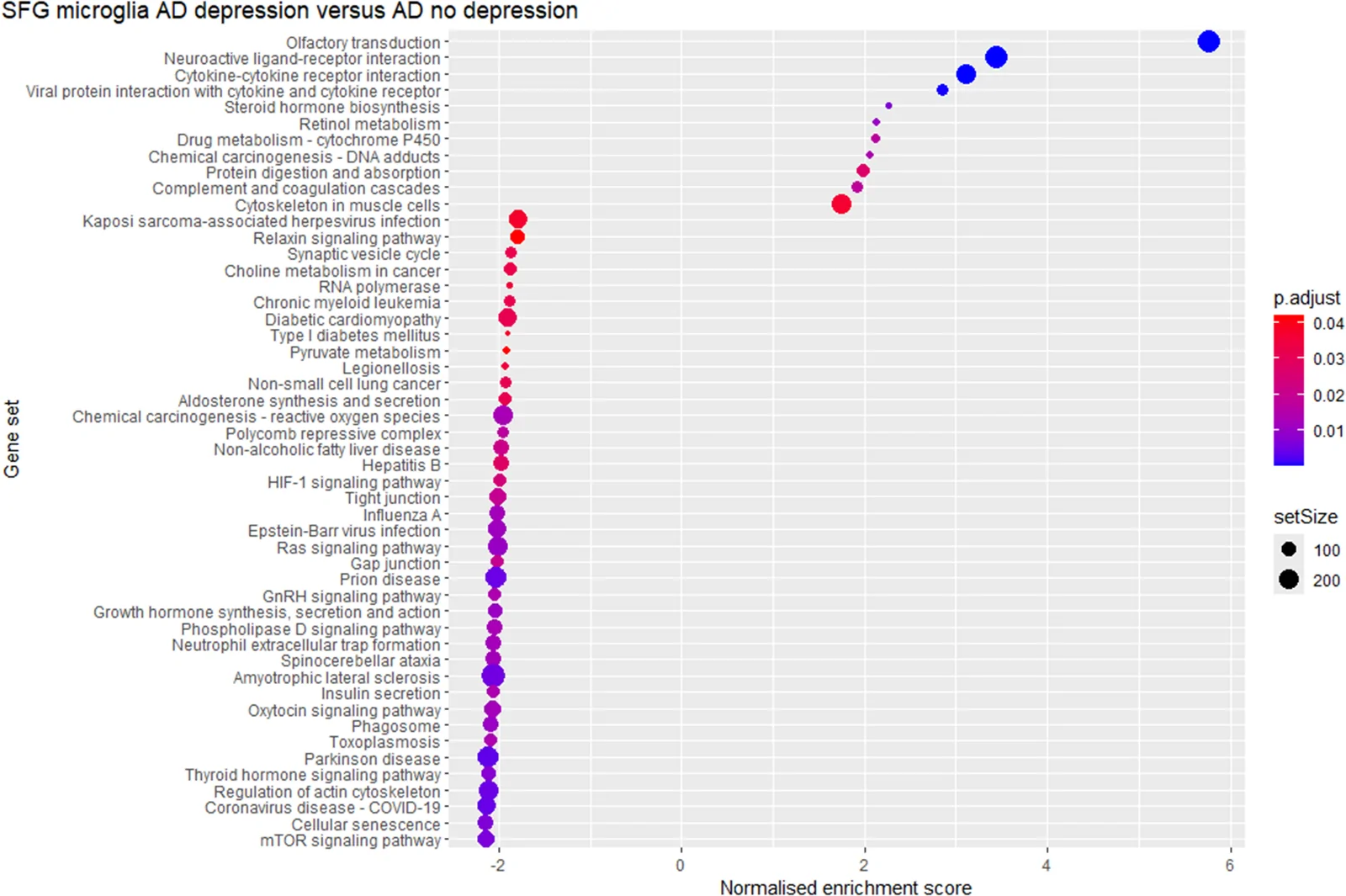
Visualization of the gene set enrichment analysis for the SFG microglial gene set, comparing all of the individuals with AD and depression to the AD without depression group. In this figure only the top 50 results are shown.

### Ains neurons

In the AIns neurons, 10 pathways were upregulated in depression compared to AD no depression. Many of these pathways were involved in general neural transmission such as glutamatergic and GABAergic signaling. The only pathway that was downregulated was olfactory transduction. Results are shown in (Figure 4 and P2: Supplemental Material 2).

**Figure 4.**
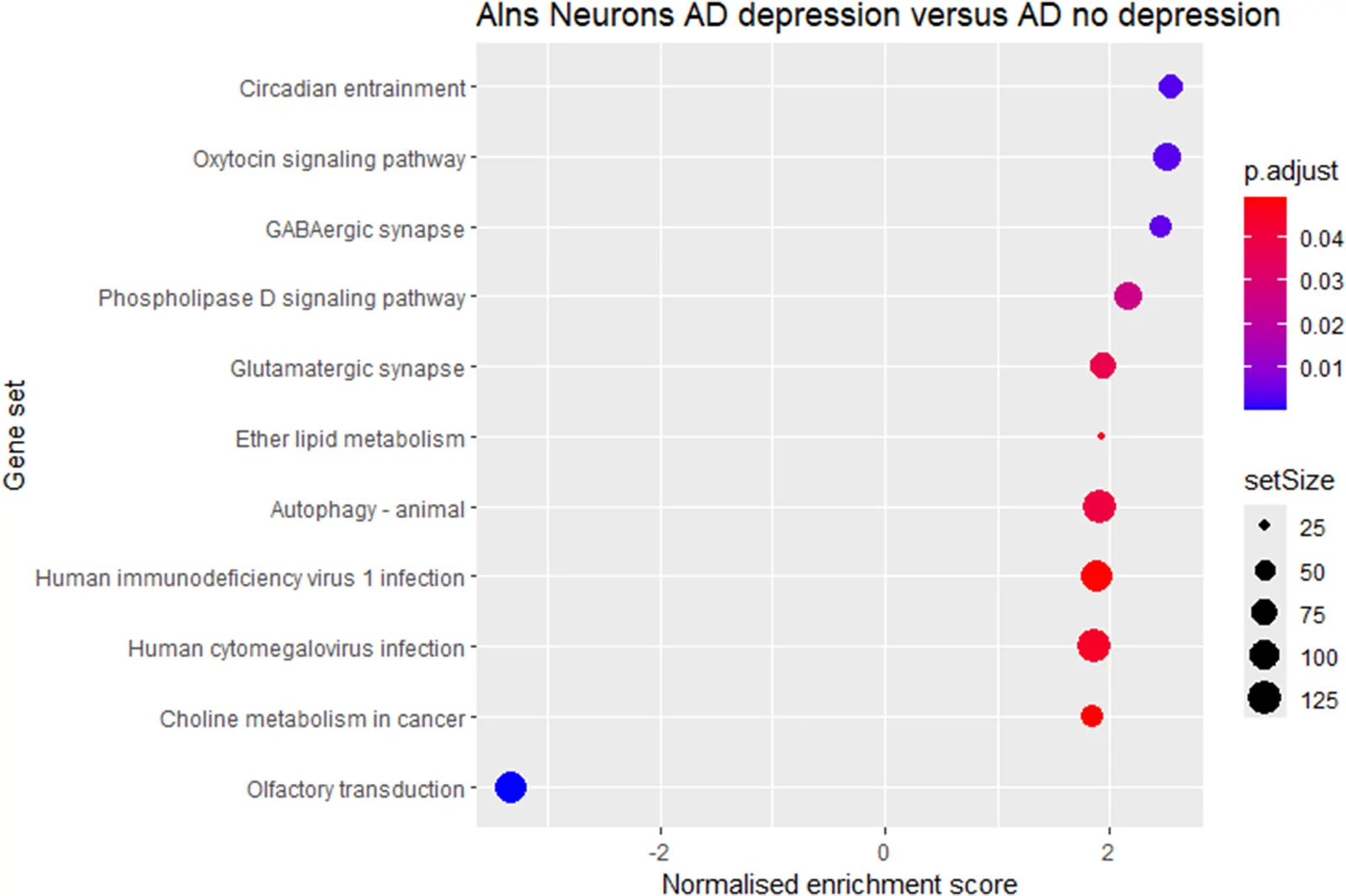
Visualization of the gene set enrichment analysis for the AIns neuronal gene set enrichment analysis, comparing all of the individuals with AD and depression to the AD without depression group.

In the PRS analysis, for the top PRS depression group versus control, no pathways were upregulated or downregulated. However, in the bottom group versus AD no depression the upregulated pathways were similar to those in the AD with depression group versus AD without depression, with 8 pathways upregulated and 4 downregulated. However, in contrast to depression versus control in our first analysis, in the bottom PRS group Morphine addiction and Dopaminergic synapse were also upregulated and several general immune response pathways were downregulated. When comparing the PRS groups, 5 pathways were upregulated in the top PRS group, 4 of which were related to immune and inflammatory responses and in the lower tertile PRS group 8 pathways were more upregulated, 5 of which were synaptic signaling or modulating pathways, including Morphine addiction and serotonergic signaling. PRS results are shown in (Supplemental Figure 9 and P2: Supplemental Material 2).

Taken together, these results suggest that in people with high PRS scores, the development of depression in AD is related to genetics that influence neuroinflammation. In AD patients with depression and low PRS scores the development of depression is probably due to environmental changes that influence neurotransmission. As those environmental changes probably include infective and other proinflammatory processes, it is noteworthy that pathways involved in inflammation and immunity are more upregulated in the high PRS group and more downregulated in the low PRS group versus AD no depression.

### SFG neurons

In superior temporal gyrus neurons, 5 pathways were enriched and none were downregulated. In contrast to the AIns, none of these was involved directly in neurotransmission. Parkinson's disease and oxidative phosphorylation were the most upregulated term, with the Alzheimer's disease term also enriched. Results are shown in (Figure 5 and P4: Supplemental Material 2). It may be relevant to the relationship between oxidative phosphorylation and depression in AD that genes involved in oxidative phosphorylation are normally downregulated in the AD brain^39,40^

**Figure 5.**
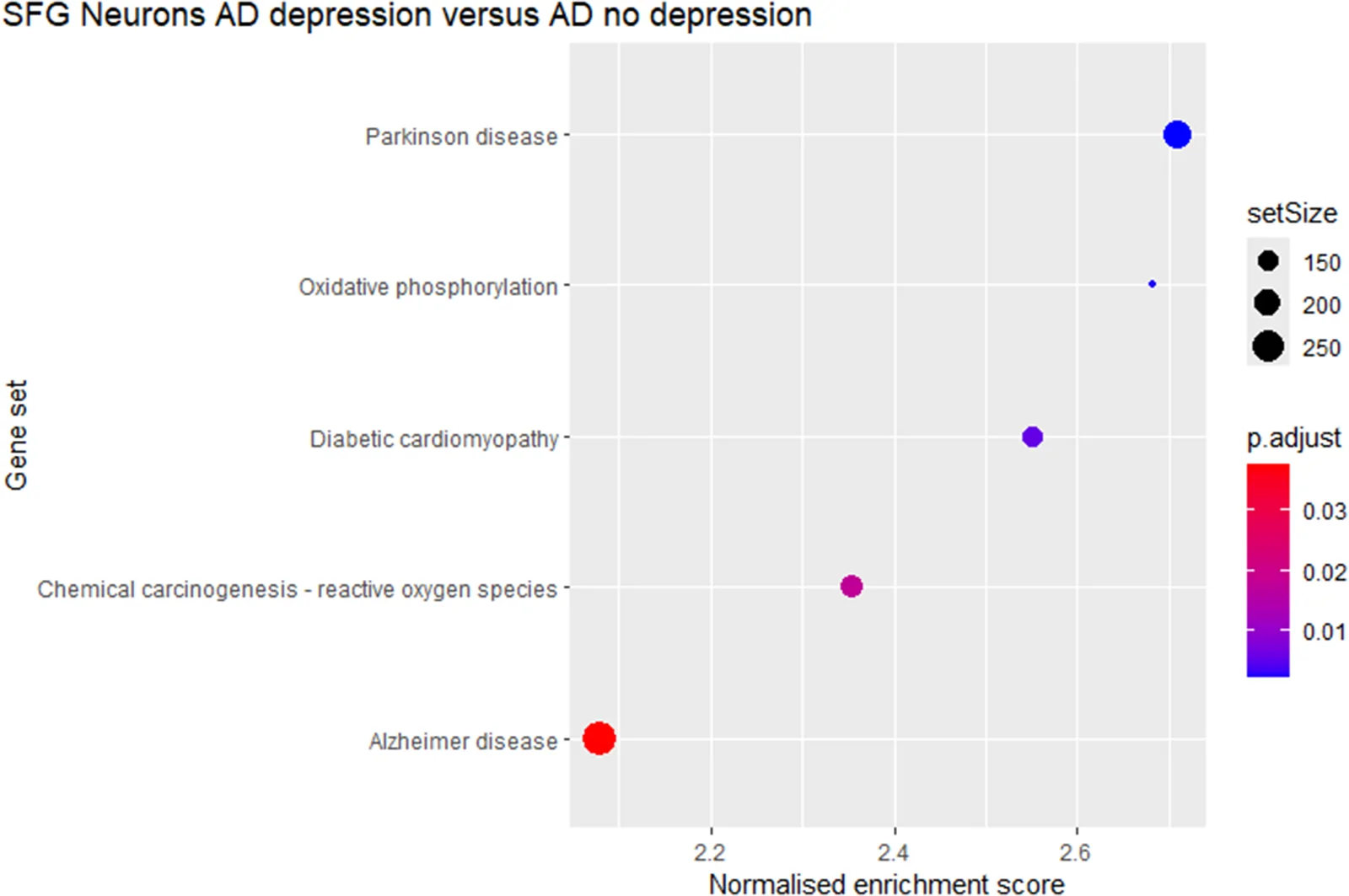
Visualization of the gene set enrichment analysis for the SFG neuronal gene set enrichment analysis, comparing all of the individuals with AD and depression to the AD without depression group.

In the PRS analysis, no pathways were either up- or downregulated in the top group compared to AD no depression, nor for the bottom PRS versus AD no depression. When comparing the upper and lower PRS groups, similarly to the AIns neurons, pathways involved in immune and inflammation were upregulated in the top PRS group, which in the SFG specifically included cytokine receptor interactions, coronavirus disease, and olfactory transduction. Only the synapse vesicle term was more upregulated in the bottom group (see Supplemental Figure 10 and P4: Supplemental Material 2).

## Discussion

In this study we aimed to examine whether gene expression in neurons or microglia was different in those with co-morbid depression and AD, compared to AD alone. We also aimed to examine whether genetic risk of depression affected these findings. Although we did not find evidence of differential gene expression for individual genes, GSEA indicated differences in the enrichment of gene expression in biological pathways in neurons and microglia. We had expected gene expression changes to be more pronounced in neurons than microglia but the opposite was true. We also found that the underlying genetic risk for depression influenced which metabolic pathways were differentially regulated between AD patients with and those without depression.

Several pathways were differentially expressed in both AIns and SFG in individuals with depression in AD, compared to people with AD who had not experienced depression. In both brain areas, there were differences in the cytokine-cytokine interaction and olfactory transduction pathways, although the direction of change was different. For example, olfactory transduction pathway was upregulated in SFG and downregulated in AIns microglia. There were no pathways in common in the neurons between the two brain areas.

Some of these pathways were also differentially regulated between the top and bottom tertiles of PRS-depression analysis. The olfactory transduction pathway was differentially expressed in the SFG microglia and neurons in both brain areas in the group at highest genetic risk of depression. Cytokine-cytokine receptor interaction and covid-19 pathways were also enriched in the neurons in both brain areas. As shown in Table 1, there were no between-group differences in antipsychotic usage, Braak Lewy body stage or anti-inflammatory medication.

Gene expression in depression in AD has been investigated in only one previous paper, which used RNAseq data and investigated one brain area only.^41^ The authors identified differentially expressed genes in their sex-stratified analysis but not in their main analysis. They reported differential expression of *CHI3L2*, a carbohydrate-binding protein with possible roles in inflammation. In individuals with and without dementia, studies of peripheral blood have identified differential expression of immune related genes^42^ and studies using brain tissue have reported differential expression of genes involved in the immune response.^43^ A related study used single nuclei RNAseq data from the ROSMAP study, from the DLPFC, but did not explicitly focus on depression in people living with dementia.^44^ The authors identified differentially expressed genes in astrocytes and neurons based on last CES-D score prior to death, but only identified changes in microglia when the average CES-D during follow-up was used. In their analysis excitatory and inhibitory neurons had the most implicated genes, unlike the current study. The authors did however comment that numbers of nuclei were lower for the less common cell types such as endothelial cells and microglia.

Our findings in relation to olfactory transduction and Parkinson's disease pathways are intriguing. It is well known that depression is more common in dementia with Lewy bodies than in AD.^45^ We made every effort to exclude individuals with neocortical Lewy body disease. We did not exclude individuals with Braak Lewy stages of ≤2 because mixed pathologies are the norm in late-onset dementia^46^ and to do so would have made our results ungeneralizable. It is possible that our findings suggest that even quite minor changes in the mix of neuropathologies in dementia could influence which behavioral and psychological symptoms of dementia are present, as shown in a recent study by Gibson et al.^18^

Our finding in the GSEA of multiple pathways related to infection or neuroinflammation being differentially expressed in microglia is intriguing and requires following up in future studies. This finding could reflect proposed links between depression, neuroinflammation, and dementia risk^47,48^ A recent paper which focused more on depression in people with AD used multiple bioinformatic methods to demonstrate that genes involved in immune regulation appeared to be involved in the pathogenesis of depression in AD.^49^ Neuroinflammation has been identified in both depression and AD in many previous studies.^50–54^ Relatively few previous studies used brain tissue, with most studying the periphery, and of the studies that did use brain tissue very few examined older adults. In our previous study of this cohort, we measured a range of cytokines in the same brain areas examined in the current study, but did not find any consistent changes in cytokines between the two brain regions.^38^ This could be because any changes in the immune system are more subtle than could be identified in a relatively small study, or do not involve the cytokines measured in our previous study. Intriguingly, the insular cortex plays an important role in interoception which has been linked to the physical manifestations of depression^55,56^ and it has also recently been identified as playing a role in immune memory.^57^

This study used brain tissue from individuals who had been extensively phenotyped during their lifetime, particularly those individuals who had been part of the Brains for Dementia Research (BDR) cohort. We used a robust definition of depression and took pains to exclude individuals with longstanding or recurrent depression that had developed more than 2 years prior to their dementia diagnosis. Likewise, we took pains to exclude individuals with depression from the AD no-depression group. Partly because of this and also because human postmortem brain tissue is a scarce resource, the numbers in this study were limited, particularly after exclusion of some cases based on our sample level QC of the microarray data. However, we were able to achieve good separation of neurons and microglia from other cell types in the tissue and, to our knowledge, this is the first study to look at cell type specific gene expression in the human brain in depression in AD.

We would point out several caveats. The first is that no individual genes were significantly differentially expressed and most fold changes were subtle. This could be because of relatively low numbers of individuals in this study, particularly in the AD no depression group. We did however include as many individuals in our AD no depression group as possible and it was not possible to increase the size of this group. We also note that our secondary analysis that subdivided the AD + depression group by the low and high PRS, was further underpowered due to both this sample size reduction in the depression group, and the increased multiple testing adjustments required due to there being 3 comparisons per analysis. Future studies should try to increase the sample size, if possible. Our GSEA results should be interpreted with caution, as it could be questioned whether these subtle gene expression changes in pathways have significant biological relevance. Further, we note that although GSEA quantifies the extent to which all the genes in a given pathway are significantly upregulated or downregulated, when compared to a significantly differentially expressed gene list in an enrichment analysis, GSEA does not necessarily factor in the magnitude of these changes, as well as the variance of these expression changes across samples in each condition. As such, we interpret that the observed GSEA pathway enrichments in our main analysis indicate changes to gene expression patterns that are more common in AD with comorbid depression, as opposed to being a hallmark gene expression difference for this phenotype. Moreover, some of the gene expression patterns in biological pathways that were indicated when we separated out the PRS conditions, might represent subtle gene expression changes that are influenced by genetics in subsets of patients.

We were unable to control for antidepressant or anti-inflammatory use in the 3 months prior to death due to missing data in some participants and cannot be certain as to whether observed expression changes were due to the depression AD phenotype or to medications such as anti-depressants which are more likely to have been administered in the depression with AD group. Although we controlled for age, Braak tangle, and age at death, most of the AD cases were in Braak stage VI; our results cannot be generalized to individuals with earlier stage AD. We included postmortem interval as a co-variate in our analyses, but as there were between group differences in postmortem interval it is possible that this could still have influenced our results.

In summary, to the best of our knowledge, this is the first study of gene expression in depression in AD in neurons and microglia, rather in than bulk tissue extracts. Although no individual gene met the threshold for differential expression our gene set enrichment analysis has identified pathways which could be involved in the pathogenesis of depression in AD. The differentially expressed gene pathways in microglia may be useful for identifying possible targets for the development of new treatments. Future studies should include a wider range of brain areas and, if possible, larger numbers of participants. The use of additional confirmatory methods such as single sample GSEA, confirmation of individual gene findings (e.g., using RT-PCR) or methods such as spatial transcriptomics could be considered to further interrogate the biology of depression in AD.

## Supplemental Material

sj-docx-1-alr-10.1177_25424823261472076 - Supplemental material for Does depression in Alzheimer's disease show evidence of differential gene expression in neurons or microglia compared to Alzheimer's disease without depression? Supplemental material, sj-docx-1-alr-10.1177_25424823261472076 for Does depression in Alzheimer's disease show evidence of differential gene expression in neurons or microglia compared to Alzheimer's disease without depression? by Lindsey I Sinclair, Mizuki Morisaki, Peter P Henley, Emma L Dempster, Clive G Ballard, Jordan T Lin, Robert MacLachlan and Seth Love in Journal of Alzheimer's Disease Reports

sj-xlsx-2-alr-10.1177_25424823261472076 - Supplemental material for Does depression in Alzheimer's disease show evidence of differential gene expression in neurons or microglia compared to Alzheimer's disease without depression? Supplemental material, sj-xlsx-2-alr-10.1177_25424823261472076 for Does depression in Alzheimer's disease show evidence of differential gene expression in neurons or microglia compared to Alzheimer's disease without depression? by Lindsey I Sinclair, Mizuki Morisaki, Peter P Henley, Emma L Dempster, Clive G Ballard, Jordan T Lin, Robert MacLachlan and Seth Love in Journal of Alzheimer's Disease Reports

## Data Availability

The datasets generated and/or analyzed during the current study are not publicly available under the terms of our material transfer agreements with the brain banks that provided tissue for this study, but are available from the corresponding author on reasonable request and with permission of the brain banks that provided tissue used in this study. Genetic data for BDR are available via the Brain Bank Database to registered Bonafide researchers. Genetic data for NACC are available via NIAGADS.
