# Supplementary material for: Does depression in Alzheimer's disease show evidence of differential gene expression in neurons or microglia compared to Alzheimer's disease without depression?
Source: J Alzheimers Dis Rep. 2026 Aug 1;10:25424823261472076. doi: 10.1177/25424823261472076 (PMC13662302; doi:10.1177/25424823261472076)
Supplement: Supplementary file 1 [file sj-docx-1-alr-10.1177_25424823261472076.docx]

**Supplemental Material**

**Does depression in Alzheimer’s disease show evidence of differential gene expression in neurons or microglia compared to Alzheimer’s disease without depression?**

### **Supplemental Figure 1.** Histogram of the distribution of the polygenic risk score for the depression in AD cohort.

### **Supplemental Table 1.** Demographic information for the individuals with AD and depression who had polygenic risk scores calculated for depression as part of this study.

|  | **Bottom tertile of genetic risk** (n=30) | **Top tertile of genetic risk** (n=29) | **Statistical evidence**  (Mann-Whitney, χ^2^ test or t-test) |
| --- | --- | --- | --- |
| Age at death (y) | 82.7 (8.8) | 84.9 (7.2) | p=0.417 |
| Postmortem interval (h) | 42.9 (28.6) | 50.7 (21.3) | p=0.110 |
| Sex  *Female*  *Male* | 14  16 | 15  14 | χ^2^=0.151, p=0.698 |
| Braak stage  III  IV  V  VI | 2  4  9  15 | 2  5  14  8 | χ^2^=5.226, p=0.389 |
| Braak LB stage  Amygdala only  0  2  unclear | 2  16  0  1 | 1  17  1  1 | χ^2^=7.03, p=0.533 |
| Clinical diagnosis  *Alzheimer’s disease*  *Depressive episode*  *Mild cognitive disorder*  *Unspecified dementia*  *Vascular dementia* | 13  0  2  4  2 | 16  1  0  4  4 | p=0.455 |
| CERAD score  *High density*  *Moderate density*  *Sparse plaques*  *No plaques* | 17  4  2  0 | 17  4  1  2 | χ^2^=4.36, p=0.498 |
| Last GDS score | 5.0 (3.4) | 13.9 (27.9) | p=0.668 |
| Last MMSE score | 9.2 (9.5) | 10.7 (8.8) | p=0.532 |

### **Supplemental Results**

Prior to each individual analysis, sample level quality control checks were performed. While checking for outliers using PCA, we observed that all the samples that were clear outliers in terms of gene expression patterns failed the positive vs negative AUC quality check, indicating that gene expression in these samples couldn’t be accurately separated from background expression. This technical difference was also attributed to lower total RNA being extracted in these samples, hence, all samples which failed this QC check were removed from the analysis.

For each of the analysis sample level QC excluded a further 9 samples from the AIC microglia analysis, 6 from AIC neurons leaving, 2 from the STG microglia, and 2 from the STG neurons.

### **Supplemental Figure 2.** PCA plot for anterior insula cortex microglia

### **Supplemental Figure 3.** PCA plot for anterior insula cortex neurons

### **Supplemental Figure 4.** Superior frontal gyrus microglia

### **Supplemental Figure 5.** Superior frontal gyrus neurons

### **Supplemental Methods: Sample level quality control checks**

Prior to the differential expression analysis, sample level QC included excluded samples that failed the AUC threshold, which measures the extent to which the array can differentiate the case condition from the control condition. We also used PCA to identify any samples which were clear outliers in terms of their gene expression patterns. For the PCA the top 2000 genes ranked by variance were used, and The PCA plots were visualized with and without the ‘weighted by variances’ transformation. Decisions to remove outliers were agreed by two authors LS and PH, which were guided by the PCA plots and other quality control metrics provided within the TAC console. Our protocol was to exclude outliers which could be attributed to technical reasons from the analysis completely, and carry out separate analysis with and without outliers where there was no technical justification, with the latter in our main results and former in the supplementary for comparison.

**Supplemental Figure 6.** Visualisation of the gene set enrichment analysis for the Anterior Insula cortex gene set enrichment analysis, comparing AD + depression to AD no depression*.*

**Supplemental Figure 7.** Visualisation of the gene set enrichment analysis for the Anterior Insula cortex microglial gene set enrichment analysis, comparing the highest tertile of PRS-depression to the lowest tertile of PRS-depression.

**Supplemental Figure 8.** Visualisation of the gene set enrichment analysis for the SFG microglial gene set enrichment analysis, comparing the highest tertile of PRS-depression to the lowest tertile of PRS-depression.

**Supplemental Figure 9.** Visualisation of the gene set enrichment analysis for the Anterior Insula cortex neuronal gene set enrichment analysis, comparing the highest tertile of PRS-depression to the lowest tertile of PRS-depression.

**Supplemental Figure 10.** Visualisation of the gene set enrichment analysis for the SFG neuronal gene set enrichment analysis, comparing the highest tertile of PRS-depression to the lowest tertile of PRS-depression.

**Supplemental Figure 11.** Rin value histograms for each cell type and brain area. Microglial values are shown in A and C, neuronal values in B and D.
